# Age and Sex Differences in Dehydroepiandrosterone Sulfate Among People With Dementia and Caregivers

**DOI:** 10.1093/geroni/igaf122.4063

**Published:** 2025-12-31

**Authors:** Wanrui Wei, Töres Theorell, Gabriella Engstrom, Azita Emami

**Affiliations:** Yale University, Orange, Connecticut, United States; Karolinska Institutet, Stockholm, Stockholms Lan, Sweden; Florida Atlantic University, Boca Raton, Florida, United States; Yale University, Orange, Connecticut, United States

## Abstract

Dehydroepiandrosterone sulfate (DHEAS) is a key adrenal hormone with neuroprotective and anti-glucocorticoid properties, yet little is known about its role in dementia and caregiving contexts. This study compared basal DHEAS levels and awakening responses between people with dementia (PWD) and their family caregivers, while examining age- and sex-specific patterns. Salivary samples were collected at awakening and 30 minutes post-awakening. Analyses included independent group comparisons, sex- and age-stratified analyses (< 65, 65–75, >75 years), and interaction modeling. Results showed that among females, PWD exhibited significantly higher basal DHEAS than caregivers after age 65, while in males, a crossover pattern emerged: PWD demonstrated higher levels before age 75, whereas caregivers showed slightly higher levels thereafter. Awakening response differences were most pronounced in younger males, with PWD displaying markedly lower responses relative to caregivers. These findings highlight distinct age- and sex-specific trajectories of DHEAS regulation in dementia. In conclusion, salivary DHEAS profiles differ between PWD and caregivers, with sex- and age-related variations. DHEAS may serve as a noninvasive biomarker of neuroendocrine function, providing insights into stress regulation and potential intervention targets for dementia care.

